# Experiences with the Scharioth Macula Lens – new hope for patients with dry macular degeneration

**Published:** 2019

**Authors:** Árpád Bereczki

**Affiliations:** * Bereczki Árpád Laser Eye Centre, Győr, Hungary

**Keywords:** age-related macular degeneration, maculopathy, low vision, visual aids, intraocular lenses

## Abstract

**Objective (aim):** Age-related macular degeneration (AMD) is the main cause of visual impairment in developed countries among the elderly. Our aim was to share our experiences with an implant designed to help improve near vision of patients with the non-exudative form of the disease.

**Methods:** 15 pseudophakic patients suffering from dry type AMD, who had been implanted with the Scharioth Macula Lens (A45SML; Medicontur, Hungary), were included in the retrospective study. Prospective visual improvement was tested preoperatively, using the method recommended by the manufacturer: improvement in near visual acuity with +2.5D compared to +6.0D addition. Follow-up period was three months. Using pre- and postoperative data, changes in near and distance visual acuity, and the correlation between the predicted and achieved improvement were evaluated.

**Results:** Preoperative corrected near vision with +2.5D and +6.0D addition was 0.17±0.07, and 0.36±0.11, respectively. Uncorrected near visual acuity 3 months postoperatively was 0.46±0.16. Predicted and actual visual improvement was 3.5 and 4.4 lines, respectively. No significant change in either the uncorrected or the best-corrected distance vision could be observed three months postoperatively. Neovascularization occurred three months postoperatively in one patient who had had stabilized wet macular degeneration before. Following treatment, the visual acuity returned to the sixth week level.

**Conclusions:** The preoperative test is a reliable tool to predict the effectiveness of the implant. Our results suggested that the SML significantly improves near visual acuity without affecting distance vision; therefore, the SML can be an effective method to ameliorate the quality of life for these patients.

**Abbreviations:** AMD = Age-Related Macular Degeneration, anti-VEGF = Anti-Vascular Endothelial Growth Factor, AREDS = Age-Related Eye Disease Study, BCDVA = Best Corrected Distance Visual Acuity, CNVA = Corrected Near Visual Acuity, ETDRS = Early Treatment Diabetic Retinopathy Study, IOL = Intraocular Lens, SML = Scharioth Macula Lens, T2.5 = Corrected Near Visual Acuity at 40 cm with an addition of +2.5 D, T6.0 = Corrected Near Visual Acuity at 15 cm with an addition of +6.0 D, UCDVA = Uncorrected Distance Visual Acuity, UCNVA = Uncorrected Near Visual Acuity

## Introduction

Age-related macular degeneration (AMD) – after cataract and glaucoma – is the third most frequent cause of visual impairment and blindness throughout the population of the world [**1**]. However, in developed countries, including Hungary, AMD has been shown to be the major aspect behind severe visual impairment and loss [**2**-**4**]. The incidence of blindness in Hungary per 100 000 inhabitants is 59.1, which means the registration of approximately 6 thousand new cases each year [**3**].

AMD is a progressive degenerative disease leading to gradual loss of vision and legal blindness; affecting the macula, which is the region of the retina responsible for sharp vision. Two types of the disease are known: the dry form represents the majority (90%) of the affected [**4**,**5**], and is characterized by the presence of so called drusen on the macula and the atrophy of the retinal pigment epithelial cells. Patients frequently experience scotoma and continuous declension in their central vision [**6**,**7**]. As AMD proceeds, neovascular (wet type) AMD develops, which means abnormal subretinal neovascularization leading to fluid accumulation and bleeding, and also subretinal and/ or intraretinal scarring, or pigment epithelium detachment can occur as a consequence [**2**].

While the introduction of anti-angiogenesis therapeutic strategies (anti-VEGF) has brought a significant advancement in the stabilization and management of wet AMD [**8**], currently there is no available treatment for dry AMD being responsible for most AMD cases. The study on age-related eye diseases (AREDS) has concluded that the regular intake of nutritional supplements containing antioxidants (vitamin C, vitamin E, beta-carotene) and zinc can reduce visual impairment, but is unable to reverse damages that have already occurred [**9**]. 

External visual aid devices designed for low vision patients have been available for decades, but their daily use is often cumbersome, and might also be stigmatizing for the patient in social situations [**6**].

These difficulties have served as a strong drive in the recent years for developing intraocular solutions. The Lipshitz intraocular telescope implant incorporates two miniature mirrors in a Cassegrain telescopic configuration. Its central element projects an enlarged image onto the retina. The implant is applied monocularly, inserted into the capsular bag through a rather large, 6.5 mm incision [**10**]. Another, subsequent approach appeared on the market, consisting of 2 intraocular lenses, based on the Galilei telescope principle. This intraocular device – similarly to the previous one – is only appropriate for implantation performed simultaneously with the cataract surgery, as one lens is inserted into the capsular bag, while the other will be located in the ciliary sulcus [**11**]. Both systems have the severe disadvantage that they are suitable only for phakic patients; however, the majority of those affected with AMD are already pseudophakic. Moreover, these implantable devices significantly affect peripheral vision and reduce the visual field, and as they are only implantable through large incisions, and the surgery can lead to a high degree of corneal astigmatism [**8**].

In 2015, a novel approach designed by Scharioth [**8**] was introduced to the market: an intraocular lens (IOL), which can be placed into the ciliary sulcus as a secondary or add-on lens. Therefore, it offers a solution for pseudophakic AMD patients, as it is possible to be implanted any time after the cataract surgery. Moreover, only a small, corneal incision (approximately 2.4 mm in diameter) is required. The add-on platform, which served as a basis for this special lens, has been used in recent years by several surgeons, and has already been proven safe (low incidence of complications) and easily implantable [**8**,**12**].

The aim of the present paper was to introduce our own experiences with the Scharioth Macula Lens (SML), paying particular attention to the near visual improvement and reading ability of the patients, and assessing the effect on distance vision. We also aimed to evaluate the method recommended by the inventor and the manufacturer for predicting the expected outcome of the SML-implantation.

## Methods

In this retrospective study, the efficacy of the Scharioth Macula Lens (A45SML, Medicontur Medical Engineering Ltd., Zsámbék, Hungary) after implantation into patients suffering from the dry type of AMD was evaluated. All pre- and postoperative examinations and surgical procedures were in accordance with the ethical standards of the Helsinki Declaration of 1975, as revised in 2000 and 2008. All data related to the patients enrolled in the study were handled and stored with care, taking into consideration the regulations of the General Data Protection Regulation (EU) 2016/ 679.

**Fig. 1:**
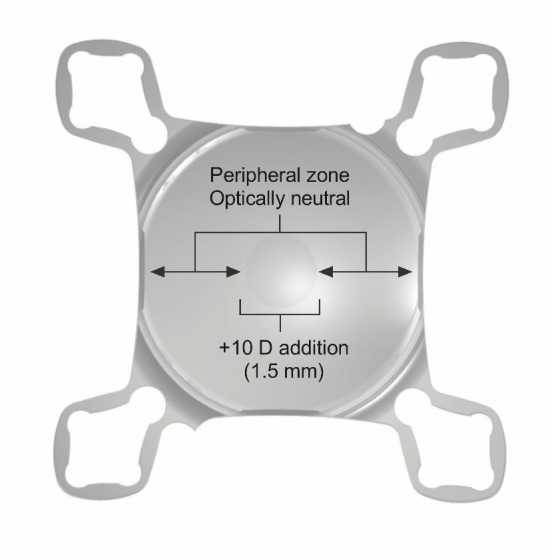
The SML. The hydrophilic acrylic IOL has a diameter of 6 mm; the lens is stabilized in the sulcus by 4 loop haptics. The peripheral zone of the lens is optically neutral, while the central 1.5 mm diameter zone offers an addition of +10.0 D

The SML is a special hydrophilic acrylic intraocular lens that can be implanted into the ciliary sulcus as an add-on IOL for pseudophakic patients. The IOL has an optically neutral peripheral region that leaves distance vision unaffected, and a central region of 1.5 mm diameter accounting for a +10.0 D addition, which results in an approximately 2.2x magnification of the image on the retina (**Fig. 1**). 

The clinical data of 15 patients were included in the analyses. The Scharioth Macula Lenses were implanted monocularly into the better seeing eye (the eye showing the better corrected distance visual acuity) between January 2015 and October 2016. All surgeries were performed at the Bereczki Laser Eye Centre in Győr, Hungary. All patients enrolled in the study had a previous cataract surgery followed by the implantation of a monofocal, non-toric intracapsular IOL. At the time of the SML-surgery, all patients were taking nutritional supplements containing antioxidants, as recommended by the AREDS examinations [**9**]. Six patients out of the 15 included in our cohort had been previously diagnosed with neovascular AMD; however, these cases were successfully stabilized by anti-VEGF (Avastin) treatment prior to the SML-implantation.

Preoperatively, a detailed eye examination was taken: uncorrected and best-corrected distance and best corrected near visual acuities (UCDVA, BCDVA, and BCNVA, respectively) were determined using standard ETDRS charts, and results were recorded as decimals. Intraocular pressure was also registered, and slit lamp and OCT examinations were performed in each case. As for the suitability of the patient for the SML-surgery, and the estimation of the expected improvement of near vision after the implantation of the lens, the testing protocol recommended by the manufacturer was performed: best corrected near visual acuity (BCNVA, the patients were wearing their prescription eyeglasses) was first tested with an addition of +2.5 D at 40 cm (T2.5), the patients wearing their own glasses or contact lenses. Then, the potential and expected reading ability after SML-implantation was simulated by adding +6.0 D and asking the patient to read at 15 cm (T6.0). 

Patients were considered suitable for the SML-implantation, where an improvement of at least 1 line (0.1 logMAR) could be observed between the results of the visual testing of T6.0, compared to T2.5.

The lenses were injected into the eye with a MedJet injector through a 2.7 clear corneal incision. The surgical procedure required not more than 10 minutes. No surgical or perioperative complication occurred in any of the cases. After the surgery, all patients were suggested to take a 10 to 15 minutes reading training on a regular, daily basis, in well-lit conditions.

Postoperative examinations were performed 1 day, 1 week, 6 weeks, and 3 months after the surgery. Visual acuities (UCDVA, BCDVA, and UCNVA) were registered and the possible correlation between preoperatively estimated and actual postoperative visual improvement was also evaluated.

Statistical analyses were performed using Microsoft Excel (Redmond, WA, USA) and GraphPad Prism version 7.04 (La Jolla, CA, USA) statistical software. Data were expressed as mean ± standard deviation (SD). All variables were tested for normal distribution using the D’Agostino & Pearson test. Depending on the results, comparisons between matching pre- and postoperative variables were performed using either the paired two-tailed t-test (in case of normal distribution) or the Wilcoxon matched-pairs signed rank test (when non-parametric test was required). Correlation analyses between pre- and postoperative reading capabilities were performed by using the non-parametric Spearman correlation analysis. P values of 0.05 or less were considered statistically significant in all cases.

## Results

Fifteen patients (8 women and 7 men) with dry type of AMD were included in the study and in the evaluations. The average age of the patients at the time of the surgery was 66.0 ± 9.6 (mean ± SD). The detailed description of data regarding each patient is summarized in **Table 1**. 

**Table 1:** Visual acuities measured preoperatively, and after the implantation of the Scharioth Macula Lens

					Preoperative					Postoperative				
Patient number	Age (years)	Sex	UCDVA	BCDVA	BCNVA +2.5 D	BCNVA +6.0 D	Estimated improvement (lines)*	UCDVA (Month 3)	BCDVA (Month 3)	UCNVA Day 1	UCNVA Week 1	UCNVA Week 6	UCNVA Month 3	Actual improvement (lines)†
1	73	M	0.40	0.50	0.16	0.40	4	0.40	0.40	0.32	0.40	0.40	0.40	4
2	61	M	0.50	0.50	0.12	0.40	5	0.50	0.50	0.32	0.40	0.40	0.50	6
3	79	F	0.50	0.60	0.20	0.50	4	0.50	0.60	0.40	0.40	0.50	0.16	-1
4	61	F	0.40	0.40	0.10	0.32	5	0.40	0.40	0.32	0.32	0.32	0.32	5
5	68	F	0.30	0.40	0.10	0.20	3	0.30	0.30	0.20	0.20	0.32	0.20	3
6	58	F	0.30	0.30	0.20	0.50	4	0.30	0.30	0.40	0.40	0.50	0.50	4
7	66	M	0.30	0.30	0.08	0.20	4	0.30	0.30	0.20	0.25	0.32	0.32	6
8	56	F	0.60	0.60	0.16	0.32	3	0.60	0.60	0.32	0.40	0.40	0.32	3
9	55	M	0.60	0.60	0.20	0.60	5	0.60	0.60	0.40	0.40	0.60	0.60	5
10	64	M	0.50	0.60	0.16	0.32	3	0.50	0.50	0.32	0.40	0.63	0.63	6
11	73	F	0.30	0.40	0.32	0.40	1	0.40	0.40	0.40	0.40	0.63	0.63	3
12	64	M	0.40	0.50	0.12	0.32	4	0.40	0.40	0.32	0.40	0.40	0.63	7
13	78	F	0.30	0.30	0.10	0.20	3	0.30	0.30	0.32	0.32	0.40	0.40	6
14	51	F	0.60	0.60	0.32	0.40	1	0.60	0.60	0.40	0.40	0.60	0.63	3
15	86	F	0.20	0.30	0.16	0.32	3	0.30	0.30	0.32	0.40	0.40	0.63	6
* Estimated improvement of vision expressed in lines: difference between the results of the preoperative measurement of BCNVA with +2.5 D and +6.0D addition. † Improvement of vision expressed in lines, compared to the preoperative measurement of BCNVA with an addition of +2.5 D.														

**Fig. 2:**
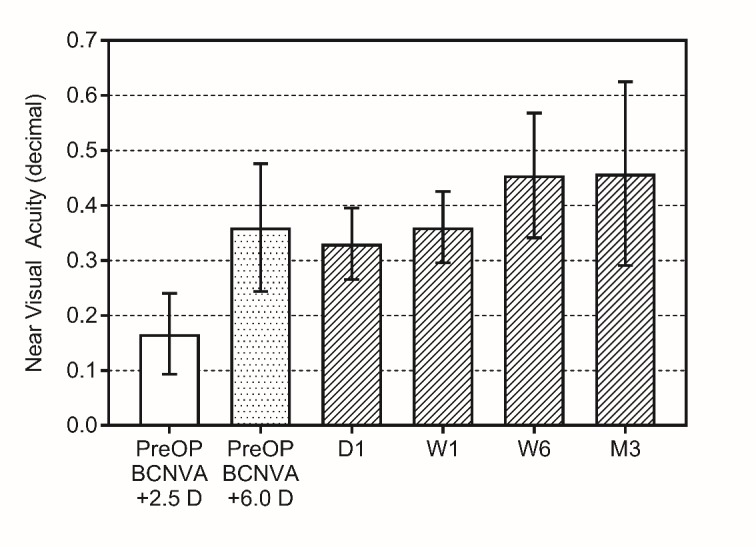
Results of the best corrected near visual acuity measurements performed preoperatively with +2.5 D (PreOP BCNVA +2.5D) and +6.0 D (PreOP BCNVA +6.0D) addition, and without any correction 1 day, 1 week, 6 weeks and 3 months after the implantation of the SML. Bars represent mean ± SD (n=15)

The pre-and postoperative results of near vision assessment are presented in **Fig. 2**. Preoperative near visual acuity corrected with +2.5 D addition (at 40 cm) had an average of 0.17 ±0.07, while with the addition of +6.0 D (at 15 cm) this value improved to 0.36 ± 0.11. After the implantation of the SML, the uncorrected near visual acuity (UCNVA) improved significantly to 0.45 ± 0.11 six weeks postoperatively (p<0.0001), and to 0.46 ± 0.16 (p<0.0001), as measured after 3 months.

**Fig. 3:**
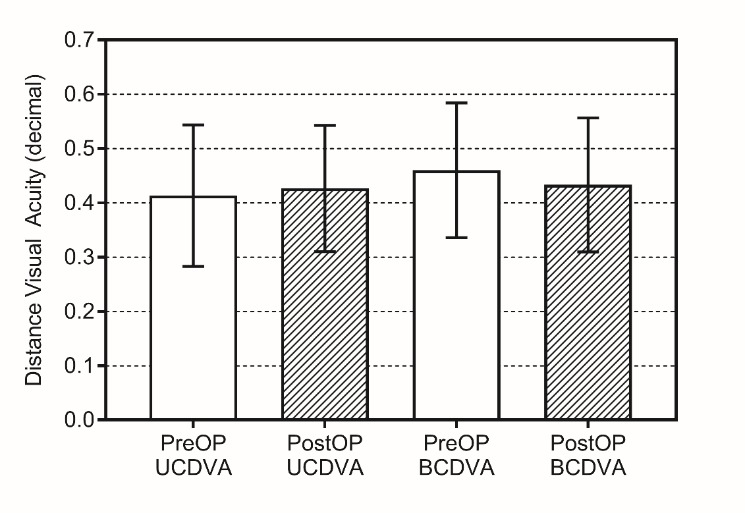
Uncorrected and best-corrected visual acuities prior to and after the surgery. Bars represent mean ± SD (n=15)

Uncorrected distance visual acuity prior to the operation showed an average of 0.41 ± 0.13, and was 0.43 ± 0.11 postoperatively (**Fig. 3**). Pre- and postoperative best corrected visual acuities were 0.46 ± 0.12 and 0.43 ± 0.12, respectively (**Fig. 3**). None of these parameters has shown any significant change in accordance with the surgery (p=0.1643 for UCDVA and p=0.5601 for BCDVA).

**Fig. 4:**
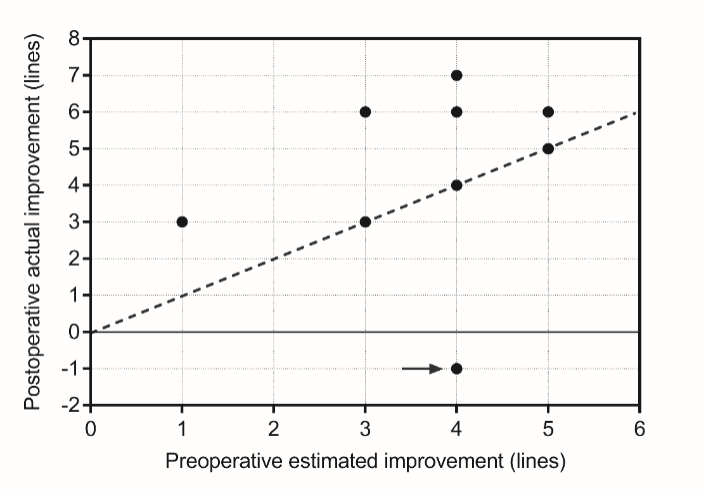
Actual improvement of near vision expressed in lines, measured 3 months postoperatively (y-axis), in correlation with the predicted improvement based on the preoperative test (x-axis). The dashed line indicates the hypothetic correlation of 1:1 between the two variables. The arrow (->) indicates the data point of the patient developing neuro vascularization 3 months postoperatively (also mentioned in the text)

According to the preoperative testing of near vision (T2.5, T6.0), an average visual improvement of 3.5 ± 1.3 lines was expected after the surgery. The actual results were even better: the patients’ postoperative near visual acuity was 4.4 ± 2.0 lines (**Table 1**). **Fig. 4** shows the single data points of the patients’ actual postoperative reading capability expressed in lines, in comparison with the preoperatively estimated values. Correlation analysis revealed that the preoperative testing protocol recommended by the manufacturer of the lens provides a reliable means for estimating the postoperative results (Spearman r=0.3126, p=0.2536).

Patient number 3 had a wet type of AMD, which was successfully stabilized prior to the operation. Three months postoperatively, neovascular AMD activated, which could be clearly observed in her visual parameters, but with the administration of anti-VEGF, vision could be restored to the level already achieved 6 weeks after the surgery (**Table 1**, **Fig. 4**).

No complications could be registered in accordance with the surgery or the SML-implantation either during the operation, or during the follow-up period.

## Discussion

In developed countries, AMD is the major cause of severe visual impairment and visual loss among the population above 50 years of age. According to the current state of medicine, no efficient treatment is known to cure the disease; however, the management of wet type AMD (affecting only a minority of the patients) using anti-VEGF therapy is promising. In the most common, dry from of the disease, drusen appear on the retina, particularly on the macula, and, as the disease progresses, affected regions continuously expand and lead to the development of scotomas in the central visual field [**7**]. 

Patients are often unable to read small print text, as a notable region of the text looks blurred, distorted, or covered by black patches of scotomas. As the absolute size of a scotoma depends on the extent of retinal damage, using magnifying devices will not help in reducing these dark regions of the visual field. Contrary, these magnifiers are able to enlarge the size of the text or the picture of interest, and this decreases the relative size of the regions affected by the scotomas. In addition, magnified letters can be also perceived by fewer sensitive light receptors located outside or around the macula easier [**13**].

The disadvantage of extraocular magnifiers is that they are often inconvenient to use, as they are usually held in the hand, and it might be cumbersome when one does the shopping, or if the patient is disabled in his/ her mobility. Trembling hands (e.g. due to Parkinson’s disease) may represent further difficulties, and performing simple daily activities like reading small print text, writing or shaving might not be feasible for the AMD-patient. Moreover, patients are prone to feel stigmatized when using their extraocular vision aid in public places or among other people. All these life situations lead to a significant decrease in the quality of life of those affected [**6**].

There was an attempt to solve the problems mentioned above by several intraocular solutions. The first devices meant serious financial burden for the patient and their implantation was usually complicated and troublesome [**8**,**10**]. Recently, smaller implants have been introduced to the market, but most of them are suitable only for phakic patients and cannot be used for patients diagnosed with maculopathy later after their cataract surgery and primary IOL implantation [**8**]. Moreover, these solutions are mostly irreversible, the devices cannot be explanted without affecting the patients’ general vision in case complications arise, or if newly developed therapies could treat macular degeneration more effectively.

The design of the Scharioth Macula Lens applied in the current study is based on a low complication rate add-on IOL platform that has been on the market for several years [**12**]. The aim of this development was to create an intraocular implant, which can ensure sufficient magnification from a relatively short reading distance, without affecting distance vision and the visual field [**8**]. The SML was designed especially for pseudophakic patients, therefore it can be implanted even into eyes that formerly underwent cataract surgery, or implantation can be combined with the cataract surgery as well. Implantation is simple to perform; the SML can be injected into the ciliary sulcus through a small, 2.7 mm diameter incision on the cornea. The surgery requires only 10 to 15 minutes, and can be easily performed as an outpatient service. The SML is independent from the type of the primary lens, and can be simply removed from the sulcus, if necessary.

Additionally, as the majority of light rays coming into the eye pass through the optically neutral peripheral region of the SML, this intraocular lens will not narrow the visual field, and will not affect distance vision adversely. Contrary, when focusing on a close object, the pupil constricts, and light rays can only pass through the central region with the +10.0 D addition. Consequently, the image projected on to the retina will be approximately 2 times magnified, which enables the patient to read texts even in smaller print.

As the SML is a rather new approach in AMD management, relatively few articles have been published on the clinical experiences [**8**]. This current paper aims to introduce our first results on 15 Hungarian patients in our practice.

Postoperative UCNVA has already reached the preoperatively estimated level (i.e. preoperative BCNVA with +6.0 D addition) 1 week after the surgery, and results 6 weeks postoperatively were even more favorable. This significant improvement proved to be stable even after 3 months following the implantation. Our results also suggested that careful patient selection and the preoperative testing protocol recommended by the manufacturer are precise and reliable tools in estimating the visual outcomes of the surgery. Regular postoperative reading training from a first unaccustomed distance of 15 cm will further enhance patients’ reading capability. We also found that patient satisfaction was in strong correlation with the patient’s motivation: motivated patients reported higher satisfaction with their vision and quality of life during the follow-up appointments.

Our results also confirmed former findings regarding the distance vision of the study population: compared to the preoperative values, neither uncorrected, nor corrected postoperative DVA values have changed after the SML implantation. 

Based on our measurements and our patients’ reports, we conclude that the Scharioth Macula Lens is a promising solution for AMD patients suffering from the dry form of the disease: a simple and minimally invasive surgery can achieve significant improvement in patients’ near vision and quality of life.

**Acknowledgements**

The results of the present article were presented in the following meetings: The annual Congress of the Hungarian Society of Intraocular Lens Implantation and Refractive Surgery (SHIOL) in Siófok, Hungary, 30th March – 1st April, 2017; and The XXXV Congress of the European Society of Cataract and Refractive Surgeons (ESCRS) in Lisbon, Portugal, 7-11th October 2017.

**Sources of funding**

The current investigation was supported by any public or any private entities, commercial or institutional supports, nor by any substantial contributions from individuals.

**Disclosures**

None.
